# Benzyl­tributyl­ammonium 6,7-dihydroxy­naphthalene-2-sulfonate

**DOI:** 10.1107/S160053680900018X

**Published:** 2009-01-17

**Authors:** Kazuya Uta, Jin Mizuguchi

**Affiliations:** a Department of Applied Physics, Graduate School of Engineering, Yokohama National University, 79-5 Tokiwadai, Hodogaya-ku, 240-8501 Yokohama, Japan

## Abstract

The title mol­ecular salt, C_19_H_34_N^+^·C_10_H_7_O_5_S^−^, is a charge-control agent used for toners in electrophotography. There are two formula units in the asymmetric unit. Both anions form inversion dimers connected by pairs of O—H⋯O hydrogen bonds between the –OH group of one anion and a sulfonic O atom of the neighboring one. The two dimers *A* and *B* are characterized by a step between the least-squares planes of the naphthalene atoms of 0.85 and 2.30 Å. Further O—H⋯O bonds link the dimers into a two-dimensional network propagating in (110) such that dimer *A* is hydrogen-bonded to four *B* units and *vice versa*. One of the *tert*-butyl chains in one of the cations is disordered over two sets of sites in a 0.56:0.44 ratio.

## Related literature

For the function of charge-control agents, see: Nash *et al.* (2001 ▶). For background and related structures, see: Mizuguchi *et al.* (2007 ▶); Uta & Mizuguchi (2009*a* ▶,*b* ▶); Sato *et al.* (2009 ▶); Uta *et al.* (2009 ▶).
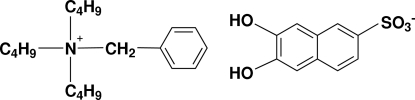


## Experimental

### 

#### Crystal data

C_19_H_34_N·C_10_H_7_O_5_S
*M* *_r_* = 515.70
Triclinic, 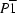

*a* = 8.6720 (2) Å
*b* = 17.1110 (3) Å
*c* = 18.8201 (4) Å
α = 86.4785 (11)°
β = 83.9245 (12)°
γ = 85.4033 (11)°
*V* = 2764.08 (10) Å^3^
*Z* = 4
Cu *K*α radiation
μ = 1.35 mm^−1^
*T* = 296.1 K
0.38 × 0.33 × 0.15 mm

#### Data collection

Rigaku R-AXIS RAPID diffractometer
Absorption correction: multi-scan (*ABSCOR*; Higashi, 1995 ▶) *T*_min_ = 0.650, *T*_max_ = 0.817
22714 measured reflections
9189 independent reflections
6913 reflections with *F* ^2^ > 2σ(*F* ^2^)
*R*_int_ = 0.038

#### Refinement

*R*[*F* ^2^ > 2σ(*F* ^2^)] = 0.068
*wR*(*F* ^2^) = 0.211
*S* = 1.12
9189 reflections
659 parameters
H-atom parameters constrained
Δρ_max_ = 0.61 e Å^−3^
Δρ_min_ = −0.47 e Å^−3^

### 

Data collection: *PROCESS-AUTO* (Rigaku, 1998 ▶); cell refinement: *PROCESS-AUTO*; data reduction: *CrystalStructure* (Rigaku/MSC, 2006 ▶); program(s) used to solve structure: *SIR2004* (Burla *et al.*, 2003 ▶); program(s) used to refine structure: *SHELXL97* (Sheldrick, 2008 ▶); molecular graphics: *ORTEPIII* (Burnett & Johnson, 1996 ▶); software used to prepare material for publication: *CrystalStructure*.

## Supplementary Material

Crystal structure: contains datablocks General, I. DOI: 10.1107/S160053680900018X/hb2892sup1.cif

Structure factors: contains datablocks I. DOI: 10.1107/S160053680900018X/hb2892Isup2.hkl

Additional supplementary materials:  crystallographic information; 3D view; checkCIF report

## Figures and Tables

**Table 1:** Hydrogen-bond geometry (Å, °)

*D*—H⋯*A*	*D*—H	H⋯*A*	*D*⋯*A*	*D*—H⋯*A*
O4—H4*O*⋯O3^i^	0.82	1.85	2.670 (3)	174
O5—H5*O*⋯O8^ii^	0.82	1.83	2.625 (3)	163
O9—H9*O*⋯O2	0.82	1.85	2.650 (3)	165
O10—H10*O*⋯O6^iii^	0.82	1.93	2.705 (3)	158

Symmetry codes: (i) 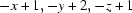; (ii) 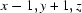; (iii) 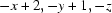.

**Fig. 1.**
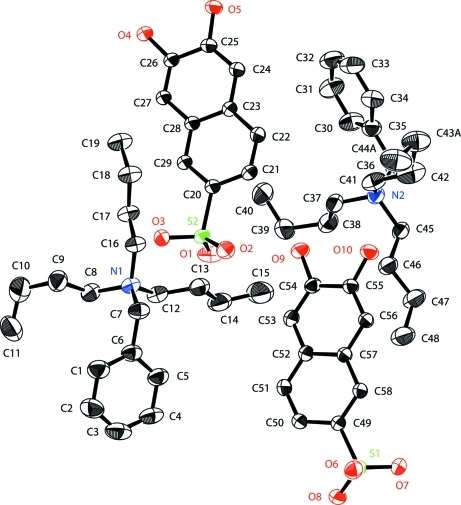
The asymmetric unit of (I), showing 30% probability displacement ellipsoids and only the major disorder components. H atoms are omitted for clarity.

**Fig. 2.**
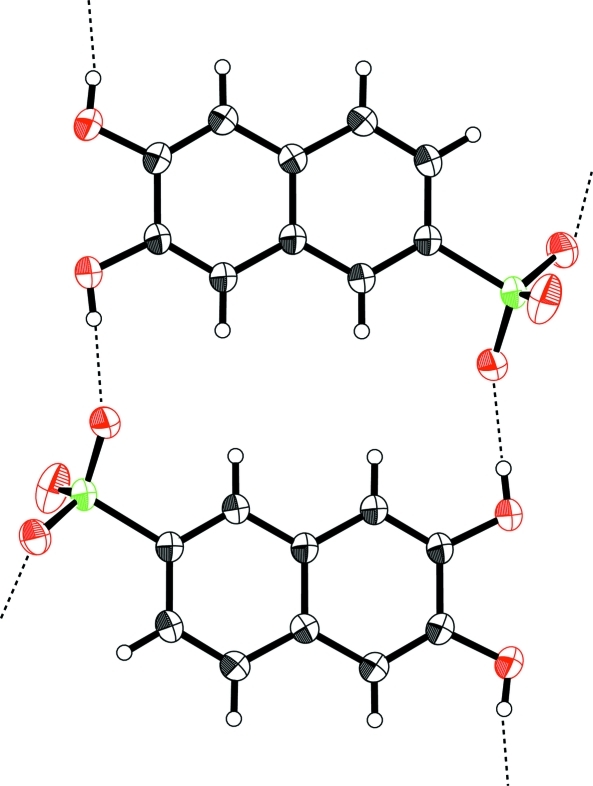
Hydrogen-bonded dimer unit A: (top view). The molecules are related by the symmetry operation (1–x, 2–y, 1–z).

**Fig. 3.**
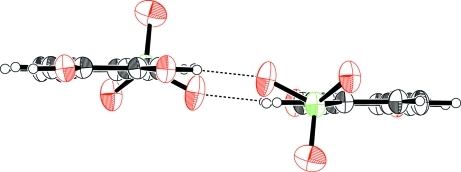
Hydrogen-bonded dimer unit A (side view).

**Fig. 4.**
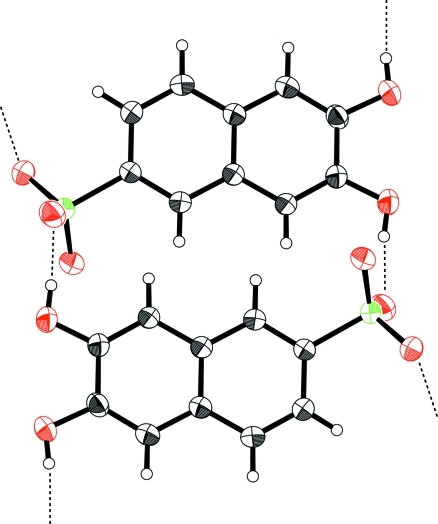
Hydrogen-bonded dimer unit B: (top view). The molecules are related by the symmetry operation (1–x, 1–y, –z).

**Fig. 5.**
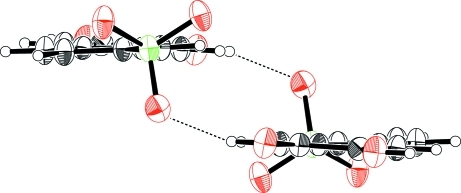
Hydrogen-bonded dimer unit B (side view).

**Fig. 6.**
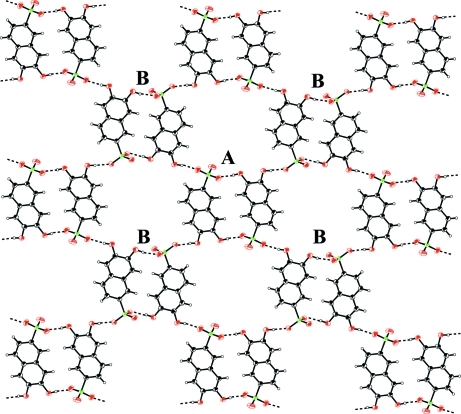
Two-dimensional hydrogen-bond network. Unit A is hydrogen-bonded to four units B and vice versa.

## supplementary crystallographic information

### Comment

Compound (I) is a charge-control-agent used for toners in electrophotography. The background of the present study has been set out in our previous paper (Uta *et al.*, 2009). We have previously investigated the crystal structure of the following four isomers in connection with the mechanism of their high melting points: benzyltributylammonium 4-hydroxynaphthalene-1-sulfonate (Mizuguchi *et al.*, 2007), benzyltributylammonium 6-hydroxynaphthalene-2-sulfonate (Uta *et al.*, 2009; benzyltributylammonium 4-hydroxynaphthalene-2-sulfonate (Uta & Mizuguchi, 2009*a*), and benzyltributylammonium 7-hydroxynaphthalene-1-sulfonate (Sato *et al.*, 2009). The melting points of these isomers are 462, 433, 451 and 439 K, respectively. Except for benzyltributylammonium 4-hydroxynaphthalene-2-sulfonate, the anions in the ammonium sulfates form chains of O—H···O intermolecular hydrogen bonds between the –OH group of one anion and the sulfonic O atom of the neighboring one. The present hydrogen-bond network is found to be responsible for the high thermal stability of these compounds. On the other hand, benzyltributylammonium 4-hydroxynaphthalene-2-sulfonate was characterized by a hydrogen-bonded dimer of the anions through O—H···O hydrogen bonding. In addition to these isomers, we have newly studied a similar ammonium sulfonate which includes two hydroxy groups in the naphthalene sulfonate as in the title compound: benzyltributylammonium 4,6-dihydroxynaphthalene-2-sulfonate which forms a two-dimensional hydrogen-bond network (Uta & Mizuguchi, 2009*b*).

There are two independent molecules in the asymmetric unit. The ions have no crystallographically imposed symmetry. Both anions form inversion dimers (A: composed of two anions of C20—C21/S2/O1—O5; B: composed of two anions of C49—C58/S1/O6—O9) through O—H···O intermoleclar hydrogen bonds (Table 1) between the –OH group of one anion and the sulfonic O atom of the neighboring one (Figs. 2 and 4).  Dimer units A and B are characterized by a step of about 0.85 and 2.30Å (Figs. 3 and 5, respectively). Then, units A and B constitute a two-dimentional O—H···O hydrogen-bond network. Unit A is hydrogen-bonded to four units B and *vice versa*, as shown in Fig. 6. The present network is quite similart to that of benzyltributylammonium 4,6-dihydroxynaphthalene-2-sulfonate (Uta & Mizuguchi, 2009*b*) and ensures a high thermal stability of compound (I) as characterized by a melting point of 478 K.

### Experimental

The title compound was obtained from Orient Chemical Industries, Ltd. and was recrystallized from an methanol solution. After 48 h, a number of colourless crystals of (I) were obtained in the form of plates.

### Refinement

C43 and C44 were found to be disordered over two sites each in a 0.56:0.44 ratio. These atoms were refined anisotropically. All H atoms were placed in geometrically idealized positions and constrained to ride on their parent atoms, with C—H = 0.93 Å (aromatic), 0.96 Å (methyl), or 0.97 Å (methylene), and O—H = 0.82 Å, *U*_iso_(H) = 1.2*U*_eq_(parent atom).

### Crystal data

**Table:**

C_19_H_34_N·C_10_H_7_O_5_S	*Z* = 4
*M**_r_* = 515.70	*F*(000) = 1112.00
Triclinic, *P*1	*D*_x_ = 1.239 Mg m^−^^3^
Hall symbol: -P 1	Cu *K*α radiation, λ = 1.54187 Å
*a* = 8.6720 (2) Å	Cell parameters from 19472 reflections
*b* = 17.1110 (3) Å	θ = 3.4–68.2°
*c* = 18.8201 (4) Å	µ = 1.35 mm^−^^1^
α = 86.4785 (11)°	*T* = 296 K
β = 83.9245 (12)°	Plate, colourless
γ = 85.4033 (11)°	0.38 × 0.33 × 0.15 mm
*V* = 2764.08 (10) Å^3^	

### Data collection

**Table:**

Rigaku R-AXIS RAPID diffractometer	6913 reflections with *F*^2^ > 2σ(*F*^2^)
Detector resolution: 10.00 pixels mm^-1^	*R*_int_ = 0.038
ω scans	θ_max_ = 68.2°
Absorption correction: multi-scan (*ABSCOR*; Higashi, 1995)	*h* = −10→10
*T*_min_ = 0.650, *T*_max_ = 0.817	*k* = −20→20
22714 measured reflections	*l* = −22→22
9189 independent reflections	

### Refinement

**Table:**

Refinement on *F*^2^	H-atom parameters constrained
*R*[*F*^2^ > 2σ(*F*^2^)] = 0.068	*w* = 1/[σ^2^(*F*_o_^2^) + (0.0999*P*)^2^ + 1.4745*P*] where *P* = (*F*_o_^2^ + 2*F*_c_^2^)/3
*wR*(*F*^2^) = 0.211	(Δ/σ)_max_ < 0.001
*S* = 1.12	Δρ_max_ = 0.61 e Å^−^^3^
9189 reflections	Δρ_min_ = −0.47 e Å^−^^3^
659 parameters	

### Special details

**Table:**

Geometry. All e.s.d.'s (except the e.s.d. in the dihedral angle between two l.s. planes) are estimated using the full covariance matrix. The cell e.s.d.'s are taken into account individually in the estimation of e.s.d.'s in distances, angles and torsion angles; correlations between e.s.d.'s in cell parameters are only used when they are defined by crystal symmetry. An approximate (isotropic) treatment of cell e.s.d.'s is used for estimating e.s.d.'s involving l.s. planes.
Refinement. Refinement of *F*^2^ against ALL reflections. The weighted *R*-factor *wR* and goodness of fit *S* are based on *F*^2^, conventional *R*-factors *R* are based on *F*, with *F* set to zero for negative *F*^2^. The threshold expression of *F*^2^ > σ(*F*^2^) is used only for calculating *R*-factors(gt) *etc*. and is not relevant to the choice of reflections for refinement. *R*-factors based on *F*^2^ are statistically about twice as large as those based on *F*, and *R*- factors based on ALL data will be even larger.

### Fractional atomic coordinates and isotropic or equivalent isotropic displacement parameters (Å^2^)

**Table:**

	*x*	*y*	*z*	*U*_iso_*/*U*_eq_	Occ. (<1)
S1	1.06446 (11)	0.30094 (5)	0.11704 (4)	0.0569 (2)	
S2	0.58851 (11)	0.80242 (5)	0.37619 (4)	0.0604 (2)	
O1	0.4657 (3)	0.75257 (16)	0.39950 (19)	0.0995 (10)	
O2	0.6913 (3)	0.77255 (16)	0.31646 (13)	0.0838 (8)	
O3	0.6737 (3)	0.82138 (16)	0.43377 (14)	0.0904 (9)	
O4	0.2201 (2)	1.21344 (13)	0.43914 (12)	0.0658 (6)	
O5	0.1545 (2)	1.23589 (13)	0.30645 (12)	0.0664 (6)	
O6	1.2260 (2)	0.31041 (15)	0.09176 (14)	0.0737 (7)	
O7	0.9746 (3)	0.28047 (14)	0.06196 (13)	0.0738 (7)	
O8	1.0478 (3)	0.24769 (14)	0.18053 (13)	0.0783 (7)	
O9	0.6544 (3)	0.73906 (14)	0.18398 (13)	0.0732 (7)	
O10	0.6797 (2)	0.71063 (13)	0.04788 (13)	0.0702 (6)	
N1	0.3094 (3)	0.70872 (17)	0.60694 (16)	0.0627 (7)	
N2	0.1740 (3)	0.78694 (18)	0.11548 (17)	0.0662 (7)	
C1	0.6084 (5)	0.5710 (2)	0.6532 (2)	0.0953 (14)	
C2	0.6471 (6)	0.4967 (3)	0.6813 (3)	0.1109 (17)	
C3	0.5748 (7)	0.4337 (3)	0.6631 (3)	0.1093 (18)	
C4	0.4656 (6)	0.4441 (2)	0.6158 (2)	0.0959 (14)	
C5	0.4267 (5)	0.5184 (2)	0.5869 (2)	0.0814 (11)	
C6	0.4952 (4)	0.5832 (2)	0.6058 (2)	0.0729 (10)	
C7	0.4579 (4)	0.6643 (2)	0.5736 (2)	0.0713 (10)	
C8	0.3072 (4)	0.7096 (2)	0.6879 (2)	0.0726 (10)	
C9	0.4293 (5)	0.7555 (2)	0.7149 (2)	0.0913 (13)	
C10	0.4066 (8)	0.7511 (4)	0.7975 (3)	0.134 (2)	
C11	0.4371 (8)	0.6768 (4)	0.8316 (3)	0.146 (2)	
C12	0.1647 (4)	0.6689 (2)	0.5951 (2)	0.0719 (10)	
C13	0.1264 (5)	0.6646 (2)	0.5198 (2)	0.0882 (12)	
C14	−0.0225 (6)	0.6144 (2)	0.5252 (3)	0.1162 (18)	
C15	−0.0912 (9)	0.6129 (3)	0.4583 (4)	0.165 (3)	
C16	0.3079 (4)	0.7909 (2)	0.5720 (2)	0.0670 (9)	
C17	0.1710 (4)	0.8469 (2)	0.5970 (2)	0.0767 (11)	
C18	0.1949 (5)	0.9283 (2)	0.5637 (2)	0.0859 (12)	
C19	0.0728 (6)	0.9900 (2)	0.5897 (3)	0.1003 (15)	
C20	0.5001 (3)	0.89379 (18)	0.34448 (16)	0.0521 (7)	
C21	0.4674 (4)	0.90556 (19)	0.27309 (17)	0.0590 (8)	
C22	0.3912 (4)	0.9741 (2)	0.25050 (17)	0.0602 (8)	
C23	0.3447 (3)	1.03487 (18)	0.29811 (16)	0.0518 (7)	
C24	0.2667 (4)	1.10708 (19)	0.27670 (17)	0.0556 (8)	
C25	0.2266 (3)	1.16439 (18)	0.32395 (17)	0.0536 (7)	
C26	0.2623 (3)	1.15214 (18)	0.39592 (16)	0.0516 (7)	
C27	0.3366 (3)	1.08346 (18)	0.41786 (16)	0.0522 (7)	
C28	0.3800 (3)	1.02278 (17)	0.37003 (16)	0.0492 (7)	
C29	0.4578 (3)	0.95124 (18)	0.39170 (16)	0.0519 (7)	
C30	−0.1203 (5)	0.9258 (2)	0.1822 (2)	0.0953 (14)	
C31	−0.1546 (6)	1.0006 (3)	0.2059 (3)	0.1098 (16)	
C32	−0.0852 (6)	1.0629 (3)	0.1713 (3)	0.1077 (17)	
C33	0.0191 (6)	1.0519 (2)	0.1126 (3)	0.1010 (15)	
C34	0.0551 (5)	0.9773 (2)	0.0875 (2)	0.0889 (12)	
C35	−0.0126 (4)	0.9128 (2)	0.1231 (2)	0.0740 (10)	
C36	0.0205 (4)	0.8301 (2)	0.0976 (2)	0.0747 (10)	
C37	0.1923 (5)	0.7879 (2)	0.1945 (2)	0.0801 (11)	
C38	0.0830 (6)	0.7426 (2)	0.2445 (2)	0.0954 (14)	
C39	0.1169 (8)	0.7492 (3)	0.3230 (2)	0.129 (2)	
C40	0.0779 (6)	0.8273 (3)	0.3513 (2)	0.1071 (16)	
C41	0.3142 (5)	0.8264 (2)	0.0779 (2)	0.0894 (12)	
C42	0.3321 (6)	0.8312 (3)	−0.0018 (3)	0.1211 (16)	
C43A	0.4120 (10)	0.8997 (5)	−0.0503 (6)	0.121 (2)	0.56
C43B	0.4814 (11)	0.8811 (7)	−0.0068 (6)	0.121 (2)	0.44
C44A	0.5731 (11)	0.8811 (7)	−0.0371 (7)	0.135 (3)	0.56
C44B	0.5504 (18)	0.8968 (10)	−0.0794 (7)	0.135 (3)	0.44
C45	0.1714 (4)	0.7040 (2)	0.0916 (2)	0.0675 (9)	
C46	0.3091 (4)	0.6478 (2)	0.1063 (2)	0.0774 (11)	
C47	0.2877 (5)	0.5676 (2)	0.0830 (3)	0.0982 (15)	
C48	0.4076 (6)	0.5046 (2)	0.1005 (3)	0.1076 (16)	
C49	0.9868 (3)	0.39446 (18)	0.14593 (16)	0.0508 (7)	
C50	0.9904 (4)	0.4139 (2)	0.21721 (17)	0.0595 (8)	
C51	0.9259 (4)	0.4844 (2)	0.23940 (17)	0.0633 (9)	
C52	0.8575 (3)	0.54087 (19)	0.19191 (16)	0.0522 (7)	
C53	0.7862 (4)	0.6136 (2)	0.21372 (17)	0.0586 (8)	
C54	0.7252 (3)	0.66777 (19)	0.16591 (17)	0.0546 (7)	
C55	0.7366 (3)	0.65187 (19)	0.09207 (17)	0.0531 (7)	
C56	0.7997 (3)	0.58110 (19)	0.07014 (16)	0.0530 (7)	
C57	0.8606 (3)	0.52288 (18)	0.11910 (16)	0.0495 (7)	
C58	0.9237 (3)	0.44814 (19)	0.09809 (16)	0.0537 (7)	
H1	0.6592	0.6139	0.6654	0.113*	
H2	0.7275	0.4898	0.7125	0.140*	
H3	0.5952	0.3831	0.6833	0.133*	
H4	0.4180	0.4014	0.6024	0.116*	
H4O	0.2464	1.2017	0.4792	0.079*	
H5	0.3546	0.5252	0.5536	0.099*	
H5O	0.1402	1.2383	0.2640	0.080*	
H7A	0.4501	0.6603	0.5231	0.088*	
H7B	0.5456	0.6952	0.5780	0.088*	
H8A	0.3194	0.6552	0.7067	0.088*	
H8B	0.2047	0.7306	0.7070	0.088*	
H9A	0.4189	0.8101	0.6972	0.112*	
H9B	0.5334	0.7340	0.6979	0.112*	
H9O	0.6547	0.7435	0.2271	0.088*	
H10A	0.4750	0.7862	0.8154	0.164*	
H10B	0.3015	0.7686	0.8135	0.164*	
H10O	0.6894	0.6964	0.0067	0.084*	
H11A	0.4229	0.6808	0.8827	0.211*	
H11B	0.5433	0.6578	0.8178	0.211*	
H11C	0.3682	0.6410	0.8177	0.211*	
H12A	0.1747	0.6153	0.6159	0.089*	
H12B	0.0759	0.6955	0.6218	0.089*	
H13A	0.2131	0.6388	0.4915	0.108*	
H13B	0.1044	0.7168	0.4992	0.108*	
H14A	0.0052	0.5618	0.5426	0.141*	
H14B	−0.1022	0.6378	0.5595	0.141*	
H15A	−0.1772	0.5817	0.4642	0.261*	
H15B	−0.0112	0.5879	0.4233	0.261*	
H15C	−0.1178	0.6643	0.4400	0.261*	
H16A	0.3099	0.7869	0.5206	0.081*	
H16B	0.4039	0.8133	0.5803	0.081*	
H17A	0.1630	0.8489	0.6488	0.093*	
H17B	0.0758	0.8293	0.5838	0.093*	
H18A	0.2960	0.9440	0.5731	0.103*	
H18B	0.1967	0.9271	0.5117	0.103*	
H19A	0.0945	1.0405	0.5657	0.154*	
H19B	0.0717	0.9938	0.6400	0.154*	
H19C	−0.0275	0.9769	0.5784	0.154*	
H21	0.4968	0.8667	0.2405	0.072*	
H22	0.3688	0.9809	0.2031	0.074*	
H24	0.2421	1.1155	0.2298	0.067*	
H27	0.3582	1.0761	0.4654	0.065*	
H29	0.4807	0.9430	0.4391	0.063*	
H30	−0.1674	0.8827	0.2063	0.114*	
H31	−0.2277	1.0097	0.2446	0.136*	
H32	−0.1071	1.1131	0.1873	0.134*	
H33	0.0679	1.0946	0.0892	0.125*	
H34	0.1238	0.9697	0.0463	0.109*	
H36A	0.0181	0.8324	0.0465	0.088*	
H36B	−0.0639	0.7991	0.1187	0.088*	
H37A	0.1820	0.8418	0.2077	0.095*	
H37B	0.2977	0.7672	0.2016	0.095*	
H38A	0.0931	0.6878	0.2334	0.117*	
H38B	−0.0230	0.7629	0.2396	0.117*	
H39A	0.0573	0.7109	0.3525	0.156*	
H39B	0.2255	0.7343	0.3261	0.156*	
H40A	0.1039	0.8278	0.3988	0.162*	
H40B	−0.0330	0.8414	0.3503	0.162*	
H40C	0.1332	0.8659	0.3211	0.162*	
H41A	0.3109	0.8796	0.0937	0.106*	
H41B	0.4081	0.7987	0.0940	0.106*	
H42A	0.2283	0.8302	−0.0163	0.145*	0.56
H42B	0.3884	0.7825	−0.0162	0.145*	0.56
H42C	0.2443	0.8594	−0.0221	0.145*	0.44
H42D	0.3542	0.7802	−0.0221	0.145*	0.44
H43A	0.3975	0.8966	−0.1004	0.145*	0.56
H43B	0.3735	0.9512	−0.0345	0.145*	0.56
H43C	0.4518	0.9306	0.0150	0.145*	0.44
H43D	0.5590	0.8528	0.0205	0.145*	0.44
H44A	0.6063	0.8293	−0.0521	0.202*	0.56
H44B	0.5826	0.8833	0.0131	0.202*	0.56
H44C	0.6369	0.9185	−0.0635	0.202*	0.56
H44D	0.6463	0.9209	−0.0783	0.202*	0.44
H44E	0.4801	0.9314	−0.1049	0.202*	0.44
H44F	0.5705	0.8483	−0.1032	0.202*	0.44
H45A	0.1622	0.7066	0.0406	0.082*	
H45B	0.0783	0.6814	0.1152	0.082*	
H46A	0.4034	0.6671	0.0802	0.095*	
H46B	0.3230	0.6454	0.1569	0.095*	
H47A	0.1867	0.5511	0.1052	0.123*	
H47B	0.2822	0.5696	0.0316	0.123*	
H48A	0.3868	0.4558	0.0836	0.163*	
H48B	0.4108	0.4994	0.1520	0.163*	
H48C	0.5084	0.5194	0.0792	0.163*	
H50	1.0393	0.3789	0.2492	0.072*	
H51	0.9281	0.4957	0.2870	0.077*	
H53	0.7803	0.6250	0.2619	0.072*	
H56	0.8051	0.5714	0.0219	0.066*	
H58	0.9196	0.4349	0.0515	0.066*	

### Atomic displacement parameters (Å^2^)

**Table:**

	*U*^11^	*U*^22^	*U*^33^	*U*^12^	*U*^13^	*U*^23^
S1	0.0768 (5)	0.0443 (4)	0.0498 (4)	0.0076 (3)	−0.0151 (3)	−0.0049 (3)
S2	0.0794 (6)	0.0453 (4)	0.0546 (4)	0.0155 (4)	−0.0090 (4)	−0.0117 (3)
O1	0.102 (2)	0.0561 (16)	0.133 (2)	0.0040 (15)	0.0016 (19)	0.0149 (16)
O2	0.105 (2)	0.0779 (17)	0.0630 (14)	0.0423 (15)	−0.0065 (13)	−0.0240 (12)
O3	0.135 (2)	0.0674 (16)	0.0721 (16)	0.0379 (16)	−0.0467 (16)	−0.0252 (13)
O4	0.0897 (17)	0.0481 (12)	0.0592 (13)	0.0168 (11)	−0.0166 (11)	−0.0132 (10)
O5	0.0882 (17)	0.0492 (12)	0.0586 (13)	0.0190 (11)	−0.0112 (11)	−0.0022 (10)
O6	0.0719 (16)	0.0735 (16)	0.0750 (16)	0.0124 (13)	−0.0089 (12)	−0.0193 (12)
O7	0.1001 (19)	0.0590 (14)	0.0665 (15)	0.0061 (13)	−0.0287 (13)	−0.0181 (11)
O8	0.128 (2)	0.0514 (13)	0.0545 (13)	0.0067 (14)	−0.0172 (14)	0.0034 (10)
O9	0.0933 (18)	0.0566 (14)	0.0659 (14)	0.0228 (13)	−0.0050 (12)	−0.0144 (11)
O10	0.0866 (17)	0.0588 (14)	0.0599 (13)	0.0240 (12)	−0.0065 (12)	−0.0014 (11)
N1	0.0625 (17)	0.0519 (16)	0.0715 (18)	−0.0035 (14)	0.0046 (13)	−0.0049 (13)
N2	0.0636 (18)	0.0604 (17)	0.0773 (19)	−0.0029 (14)	−0.0155 (14)	−0.0125 (14)
C1	0.089 (3)	0.078 (3)	0.117 (3)	0.014 (2)	−0.020 (2)	−0.010 (2)
C2	0.109 (4)	0.098 (3)	0.121 (4)	0.032 (3)	−0.025 (3)	−0.002 (3)
C3	0.119 (4)	0.077 (3)	0.119 (4)	0.032 (3)	0.008 (3)	0.012 (2)
C4	0.107 (3)	0.056 (2)	0.117 (3)	0.016 (2)	0.006 (3)	−0.010 (2)
C5	0.087 (2)	0.064 (2)	0.090 (2)	0.011 (2)	0.000 (2)	−0.013 (2)
C6	0.074 (2)	0.061 (2)	0.078 (2)	0.0080 (19)	0.0072 (19)	−0.0026 (18)
C7	0.064 (2)	0.062 (2)	0.082 (2)	0.0061 (18)	0.0124 (18)	−0.0014 (18)
C8	0.080 (2)	0.064 (2)	0.072 (2)	−0.009 (2)	0.0058 (19)	−0.0076 (18)
C9	0.107 (3)	0.087 (3)	0.083 (2)	−0.023 (2)	−0.011 (2)	−0.006 (2)
C10	0.169 (6)	0.125 (5)	0.118 (4)	−0.029 (4)	−0.039 (4)	−0.014 (3)
C11	0.175 (6)	0.142 (5)	0.134 (5)	−0.025 (4)	−0.077 (4)	0.020 (4)
C12	0.065 (2)	0.055 (2)	0.094 (2)	−0.0046 (18)	0.0016 (19)	−0.0078 (19)
C13	0.097 (3)	0.062 (2)	0.108 (3)	0.001 (2)	−0.028 (2)	−0.010 (2)
C14	0.130 (4)	0.064 (2)	0.162 (5)	−0.008 (2)	−0.048 (4)	−0.007 (3)
C15	0.172 (6)	0.099 (4)	0.241 (9)	−0.007 (4)	−0.108 (6)	−0.006 (5)
C16	0.066 (2)	0.0498 (19)	0.082 (2)	−0.0026 (17)	0.0060 (18)	−0.0016 (17)
C17	0.076 (2)	0.054 (2)	0.097 (2)	0.0012 (19)	0.003 (2)	−0.0078 (19)
C18	0.082 (2)	0.061 (2)	0.111 (3)	0.002 (2)	0.005 (2)	−0.002 (2)
C19	0.110 (3)	0.063 (2)	0.123 (3)	0.009 (2)	0.000 (3)	−0.009 (2)
C20	0.0615 (19)	0.0443 (17)	0.0499 (16)	0.0054 (14)	−0.0075 (14)	−0.0057 (13)
C21	0.074 (2)	0.0485 (18)	0.0543 (18)	0.0084 (16)	−0.0073 (15)	−0.0139 (14)
C22	0.078 (2)	0.0541 (19)	0.0474 (17)	0.0077 (17)	−0.0095 (15)	−0.0081 (14)
C23	0.0612 (19)	0.0469 (17)	0.0473 (16)	0.0009 (14)	−0.0068 (13)	−0.0060 (13)
C24	0.067 (2)	0.0483 (17)	0.0503 (17)	0.0040 (15)	−0.0086 (14)	−0.0015 (13)
C25	0.0591 (19)	0.0440 (17)	0.0568 (18)	0.0039 (14)	−0.0082 (14)	−0.0003 (13)
C26	0.0606 (19)	0.0427 (16)	0.0505 (16)	0.0046 (14)	−0.0041 (13)	−0.0083 (13)
C27	0.0626 (19)	0.0447 (16)	0.0485 (16)	0.0063 (14)	−0.0080 (14)	−0.0062 (13)
C28	0.0548 (18)	0.0424 (16)	0.0496 (16)	0.0036 (14)	−0.0048 (13)	−0.0052 (12)
C29	0.0625 (19)	0.0439 (16)	0.0491 (16)	0.0053 (14)	−0.0085 (13)	−0.0074 (13)
C30	0.086 (3)	0.071 (2)	0.123 (3)	0.009 (2)	0.009 (2)	−0.010 (2)
C31	0.110 (4)	0.084 (3)	0.132 (4)	0.011 (3)	0.004 (3)	−0.025 (3)
C32	0.101 (3)	0.066 (3)	0.158 (5)	0.013 (2)	−0.028 (3)	−0.019 (3)
C33	0.095 (3)	0.061 (2)	0.146 (4)	0.000 (2)	−0.021 (3)	0.006 (2)
C34	0.088 (3)	0.071 (2)	0.104 (3)	0.005 (2)	−0.010 (2)	0.006 (2)
C35	0.070 (2)	0.061 (2)	0.093 (2)	0.0008 (19)	−0.018 (2)	−0.005 (2)
C36	0.068 (2)	0.072 (2)	0.086 (2)	0.0037 (19)	−0.0206 (19)	−0.011 (2)
C37	0.087 (2)	0.072 (2)	0.086 (2)	0.001 (2)	−0.027 (2)	−0.023 (2)
C38	0.128 (4)	0.075 (2)	0.083 (2)	−0.006 (2)	−0.007 (2)	−0.018 (2)
C39	0.206 (6)	0.100 (3)	0.080 (3)	0.035 (4)	−0.036 (3)	−0.013 (2)
C40	0.120 (4)	0.109 (3)	0.090 (3)	0.016 (3)	−0.004 (2)	−0.029 (2)
C41	0.072 (2)	0.062 (2)	0.132 (3)	−0.010 (2)	0.003 (2)	−0.013 (2)
C42	0.123 (3)	0.091 (3)	0.136 (3)	−0.010 (2)	0.045 (3)	−0.001 (2)
C43A	0.116 (4)	0.094 (4)	0.141 (5)	0.000 (3)	0.026 (3)	0.011 (3)
C43B	0.116 (4)	0.094 (4)	0.141 (5)	0.000 (3)	0.026 (3)	0.011 (3)
C44A	0.115 (4)	0.115 (6)	0.159 (7)	−0.004 (4)	0.030 (5)	0.041 (6)
C44B	0.115 (4)	0.115 (6)	0.159 (7)	−0.004 (4)	0.030 (5)	0.041 (6)
C45	0.071 (2)	0.060 (2)	0.074 (2)	−0.0047 (18)	−0.0136 (18)	−0.0160 (17)
C46	0.074 (2)	0.065 (2)	0.096 (2)	0.001 (2)	−0.016 (2)	−0.018 (2)
C47	0.091 (3)	0.068 (2)	0.140 (4)	0.002 (2)	−0.026 (2)	−0.028 (2)
C48	0.113 (4)	0.070 (2)	0.140 (4)	0.003 (2)	−0.019 (3)	−0.016 (2)
C49	0.0613 (19)	0.0457 (17)	0.0454 (15)	0.0009 (14)	−0.0073 (13)	−0.0057 (12)
C50	0.077 (2)	0.0514 (18)	0.0494 (17)	0.0053 (16)	−0.0125 (15)	−0.0016 (14)
C51	0.086 (2)	0.061 (2)	0.0434 (16)	0.0049 (18)	−0.0112 (16)	−0.0067 (14)
C52	0.0586 (19)	0.0503 (18)	0.0473 (16)	−0.0008 (15)	−0.0040 (13)	−0.0058 (13)
C53	0.070 (2)	0.0566 (19)	0.0483 (17)	0.0015 (16)	−0.0030 (14)	−0.0108 (14)
C54	0.0562 (19)	0.0487 (18)	0.0573 (18)	0.0043 (15)	0.0000 (14)	−0.0099 (14)
C55	0.0569 (18)	0.0491 (17)	0.0521 (17)	0.0045 (14)	−0.0063 (13)	−0.0028 (13)
C56	0.0609 (19)	0.0517 (18)	0.0449 (16)	0.0076 (15)	−0.0069 (13)	−0.0048 (13)
C57	0.0529 (17)	0.0491 (17)	0.0457 (15)	0.0014 (14)	−0.0051 (12)	−0.0041 (12)
C58	0.066 (2)	0.0505 (18)	0.0443 (16)	0.0042 (15)	−0.0085 (14)	−0.0058 (13)

### Geometric parameters (Å, °)

**Table:**

S1—O6	1.450 (2)	C4—H4	0.929
S1—O7	1.437 (2)	C5—H5	0.927
S1—O8	1.459 (2)	C7—H7A	0.967
S1—C49	1.779 (3)	C7—H7B	0.974
S2—O1	1.433 (3)	C8—H8A	0.977
S2—O2	1.451 (2)	C8—H8B	0.972
S2—O3	1.441 (3)	C9—H9A	0.973
S2—C20	1.783 (3)	C9—H9B	0.976
O4—C26	1.370 (3)	C10—H10A	0.976
O5—C25	1.366 (3)	C10—H10B	0.959
O9—C54	1.367 (3)	C11—H11A	0.962
O10—C55	1.361 (3)	C11—H11B	0.966
N1—C7	1.536 (4)	C11—H11C	0.955
N1—C8	1.523 (5)	C12—H12A	0.974
N1—C12	1.517 (5)	C12—H12B	0.971
N1—C16	1.514 (4)	C13—H13A	0.967
N2—C36	1.529 (4)	C13—H13B	0.965
N2—C37	1.514 (5)	C14—H14A	0.960
N2—C41	1.525 (5)	C14—H14B	0.974
N2—C45	1.517 (4)	C15—H15A	0.945
C1—C2	1.377 (7)	C15—H15B	0.994
C1—C6	1.391 (6)	C15—H15C	0.945
C2—C3	1.368 (8)	C16—H16A	0.972
C3—C4	1.361 (8)	C16—H16B	0.975
C4—C5	1.382 (6)	C17—H17A	0.973
C5—C6	1.381 (6)	C17—H17B	0.962
C6—C7	1.503 (5)	C18—H18A	0.975
C8—C9	1.514 (6)	C18—H18B	0.978
C9—C10	1.544 (8)	C19—H19A	0.972
C10—C11	1.407 (9)	C19—H19B	0.950
C12—C13	1.497 (6)	C19—H19C	0.964
C13—C14	1.598 (7)	C21—H21	0.934
C14—C15	1.451 (10)	C22—H22	0.931
C16—C17	1.518 (5)	C24—H24	0.929
C17—C18	1.512 (5)	C27—H27	0.932
C18—C19	1.500 (6)	C29—H29	0.934
C20—C21	1.402 (4)	C30—H30	0.940
C20—C29	1.368 (4)	C31—H31	0.926
C21—C22	1.367 (4)	C32—H32	0.925
C22—C23	1.420 (4)	C33—H33	0.937
C23—C24	1.418 (4)	C34—H34	0.935
C23—C28	1.418 (4)	C36—H36A	0.963
C24—C25	1.364 (4)	C36—H36B	0.975
C25—C26	1.421 (4)	C37—H37A	0.965
C26—C27	1.357 (4)	C37—H37B	0.973
C27—C28	1.418 (4)	C38—H38A	0.968
C28—C29	1.409 (4)	C38—H38B	0.968
C30—C31	1.381 (7)	C39—H39A	0.976
C30—C35	1.392 (6)	C39—H39B	0.963
C31—C32	1.363 (7)	C40—H40A	0.946
C32—C33	1.364 (8)	C40—H40B	0.975
C33—C34	1.390 (6)	C40—H40C	0.969
C34—C35	1.393 (6)	C41—H41A	0.972
C35—C36	1.517 (5)	C41—H41B	0.977
C37—C38	1.491 (6)	C42—H42A	0.970
C38—C39	1.549 (7)	C42—H42B	0.970
C39—C40	1.470 (7)	C42—H42C	0.970
C41—C42	1.488 (7)	C42—H42D	0.970
C42—C43A	1.598 (10)	C43A—H43A	0.970
C42—C43B	1.598 (13)	C43A—H43B	0.970
C43A—C44A	1.451 (13)	C43B—H43C	0.970
C43B—C44B	1.451 (17)	C43B—H43D	0.970
C45—C46	1.511 (5)	C44A—H44A	0.960
C46—C47	1.496 (6)	C44A—H44B	0.960
C47—C48	1.485 (6)	C44A—H44C	0.960
C49—C50	1.406 (4)	C44B—H44D	0.960
C49—C58	1.370 (4)	C44B—H44E	0.960
C50—C51	1.360 (4)	C44B—H44F	0.960
C51—C52	1.413 (4)	C45—H45A	0.970
C52—C53	1.409 (4)	C45—H45B	0.975
C52—C57	1.420 (4)	C46—H46A	0.978
C53—C54	1.367 (4)	C46—H46B	0.971
C54—C55	1.424 (4)	C47—H47A	0.984
C55—C56	1.358 (4)	C47—H47B	0.973
C56—C57	1.423 (4)	C48—H48A	0.949
C57—C58	1.413 (4)	C48—H48B	0.971
O4—H4O	0.820	C48—H48C	0.969
O5—H5O	0.820	C50—H50	0.931
O9—H9O	0.820	C51—H51	0.931
O10—H10O	0.820	C53—H53	0.934
C1—H1	0.938	C56—H56	0.928
C2—H2	0.954	C58—H58	0.924
C3—H3	0.935		
			
O2···O9	2.650 (3)	O6···H11B^viii^	2.864
O3···O4^i^	2.670 (3)	O6···H44A^iii^	2.797
O4···O3^i^	2.670 (3)	O6···H48A^vi^	2.936
O5···O8^ii^	2.625 (3)	O6···H56^iii^	2.871
O6···O10^iii^	2.705 (3)	O7···H36A^ix^	2.887
O8···O5^iv^	2.625 (3)	O7···H42A^ix^	2.911
O9···O2	2.650 (3)	O7···H45A^ix^	2.360
O10···O6^iii^	2.705 (3)	O8···H5O^iv^	1.830
S1···H10O^iii^	2.989	O8···H8B^v^	2.897
O1···H3^v^	2.985	O8···H24^iv^	2.877
O1···H4^v^	2.745	O8···H32^iv^	2.748
O1···H7A	2.727	O9···H21	2.696
O1···H16A	2.598	O9···H36B^vi^	2.854
O1···H39B	2.666	O9···H41B	2.945
O2···H9O	1.849	O10···H41B	2.780
O2···H30^vi^	2.950	O10···H43D	2.614
O2···H38B^vi^	2.731	O10···H44A	2.765
O2···H40B^vi^	2.893	O10···H46A	2.567
O2···H53	2.804	C15···H39A	2.798
O3···H4O^i^	1.853	C25···H9B^i^	2.799
O3···H27^i^	2.641	C25···H17B^vii^	2.986
O3···H40B^vi^	2.878	C26···H17B^vii^	2.911
O4···H7B^i^	2.643	C27···H16B^i^	2.975
O4···H14B^vii^	2.666	C27···H19C^vii^	2.941
O4···H17B^vii^	2.807	C33···H44D^x^	2.973
O5···H9B^i^	2.787	C44B···H10A^xi^	2.976
O5···H12B^vii^	2.538	C55···H46A	2.912
O5···H17B^vii^	2.950	C56···H48C	2.800
O5···H50^ii^	2.768	C57···H47A^vi^	2.889
O6···H10O^iii^	1.927		
			
O6—S1—O7	113.17 (15)	C14—C13—H13A	110.7
O6—S1—O8	112.23 (16)	C14—C13—H13B	111.4
O6—S1—C49	105.71 (15)	H13A—C13—H13B	109.3
O7—S1—O8	112.32 (16)	C13—C14—H14A	109.3
O7—S1—C49	107.42 (15)	C13—C14—H14B	108.5
O8—S1—C49	105.34 (14)	C15—C14—H14A	109.9
O1—S2—O2	112.94 (18)	C15—C14—H14B	106.9
O1—S2—O3	112.85 (19)	H14A—C14—H14B	108.2
O1—S2—C20	107.19 (16)	C14—C15—H15A	110.4
O2—S2—O3	111.74 (17)	C14—C15—H15B	107.3
O2—S2—C20	106.23 (14)	C14—C15—H15C	111.0
O3—S2—C20	105.25 (15)	H15A—C15—H15B	107.9
C7—N1—C8	111.3 (2)	H15A—C15—H15C	112.2
C7—N1—C12	111.7 (2)	H15B—C15—H15C	107.9
C7—N1—C16	105.9 (2)	N1—C16—H16A	108.4
C8—N1—C12	104.8 (2)	N1—C16—H16B	108.1
C8—N1—C16	111.7 (2)	C17—C16—H16A	108.6
C12—N1—C16	111.5 (2)	C17—C16—H16B	108.8
C36—N2—C37	111.3 (2)	H16A—C16—H16B	106.8
C36—N2—C41	111.9 (2)	C16—C17—H17A	109.7
C36—N2—C45	106.1 (2)	C16—C17—H17B	110.2
C37—N2—C41	105.0 (3)	C18—C17—H17A	108.9
C37—N2—C45	111.8 (2)	C18—C17—H17B	109.7
C41—N2—C45	110.8 (2)	H17A—C17—H17B	108.6
C2—C1—C6	120.6 (4)	C17—C18—H18A	109.8
C1—C2—C3	120.6 (5)	C17—C18—H18B	109.2
C2—C3—C4	119.8 (5)	C19—C18—H18A	108.3
C3—C4—C5	120.0 (4)	C19—C18—H18B	108.6
C4—C5—C6	121.2 (4)	H18A—C18—H18B	106.6
C1—C6—C5	117.7 (3)	C18—C19—H19A	109.7
C1—C6—C7	119.9 (3)	C18—C19—H19B	110.2
C5—C6—C7	122.2 (3)	C18—C19—H19C	109.5
N1—C7—C6	116.1 (3)	H19A—C19—H19B	109.3
N1—C8—C9	115.9 (3)	H19A—C19—H19C	108.1
C8—C9—C10	108.4 (4)	H19B—C19—H19C	110.0
C9—C10—C11	116.4 (5)	C20—C21—H21	120.5
N1—C12—C13	117.9 (3)	C22—C21—H21	119.3
C12—C13—C14	105.6 (3)	C21—C22—H22	119.3
C13—C14—C15	113.9 (4)	C23—C22—H22	119.5
N1—C16—C17	115.8 (2)	C23—C24—H24	119.6
C16—C17—C18	109.6 (3)	C25—C24—H24	119.4
C17—C18—C19	114.1 (3)	C26—C27—H27	119.1
S2—C20—C21	120.7 (2)	C28—C27—H27	119.8
S2—C20—C29	119.0 (2)	C20—C29—H29	119.6
C21—C20—C29	120.2 (2)	C28—C29—H29	119.4
C20—C21—C22	120.2 (2)	C31—C30—H30	120.8
C21—C22—C23	121.2 (3)	C35—C30—H30	118.9
C22—C23—C24	123.0 (2)	C30—C31—H31	120.7
C22—C23—C28	118.2 (2)	C32—C31—H31	118.6
C24—C23—C28	118.8 (2)	C31—C32—H32	120.8
C23—C24—C25	121.0 (2)	C33—C32—H32	119.1
O5—C25—C24	124.0 (2)	C32—C33—H33	120.2
O5—C25—C26	116.0 (2)	C34—C33—H33	119.4
C24—C25—C26	120.1 (2)	C33—C34—H34	120.7
O4—C26—C25	116.0 (2)	C35—C34—H34	119.2
O4—C26—C27	123.9 (2)	N2—C36—H36A	108.5
C25—C26—C27	120.1 (2)	N2—C36—H36B	108.0
C26—C27—C28	121.2 (2)	C35—C36—H36A	108.3
C23—C28—C27	118.8 (2)	C35—C36—H36B	107.8
C23—C28—C29	119.2 (2)	H36A—C36—H36B	107.5
C27—C28—C29	122.0 (2)	N2—C37—H37A	108.3
C20—C29—C28	121.0 (2)	N2—C37—H37B	107.7
C31—C30—C35	120.3 (4)	C38—C37—H37A	108.3
C30—C31—C32	120.7 (5)	C38—C37—H37B	107.8
C31—C32—C33	120.1 (5)	H37A—C37—H37B	107.4
C32—C33—C34	120.4 (4)	C37—C38—H38A	110.2
C33—C34—C35	120.1 (4)	C37—C38—H38B	109.8
C30—C35—C34	118.3 (3)	C39—C38—H38A	109.0
C30—C35—C36	119.1 (3)	C39—C38—H38B	108.8
C34—C35—C36	122.5 (3)	H38A—C38—H38B	108.3
N2—C36—C35	116.5 (3)	C38—C39—H39A	107.5
N2—C37—C38	116.9 (3)	C38—C39—H39B	108.1
C37—C38—C39	110.7 (4)	C40—C39—H39A	109.0
C38—C39—C40	114.8 (4)	C40—C39—H39B	109.5
N2—C41—C42	117.6 (4)	H39A—C39—H39B	107.7
C41—C42—C43A	124.7 (5)	C39—C40—H40A	110.7
C41—C42—C43B	93.5 (5)	C39—C40—H40B	109.7
C42—C43A—C44A	100.0 (7)	C39—C40—H40C	109.5
C42—C43B—C44B	113.8 (10)	H40A—C40—H40B	109.4
N2—C45—C46	116.6 (3)	H40A—C40—H40C	109.9
C45—C46—C47	110.8 (3)	H40B—C40—H40C	107.6
C46—C47—C48	116.5 (4)	N2—C41—H41A	108.5
S1—C49—C50	120.6 (2)	N2—C41—H41B	108.2
S1—C49—C58	119.6 (2)	C42—C41—H41A	107.6
C50—C49—C58	119.8 (2)	C42—C41—H41B	107.9
C49—C50—C51	120.2 (3)	H41A—C41—H41B	106.6
C50—C51—C52	121.7 (3)	C41—C42—H42A	106.2
C51—C52—C53	123.0 (2)	C41—C42—H42B	106.1
C51—C52—C57	118.2 (2)	C41—C42—H42C	113.0
C53—C52—C57	118.9 (2)	C41—C42—H42D	113.0
C52—C53—C54	121.4 (2)	C43A—C42—H42A	106.1
O9—C54—C53	124.0 (2)	C43A—C42—H42B	106.2
O9—C54—C55	116.2 (2)	C43B—C42—H42C	113.0
C53—C54—C55	119.9 (2)	C43B—C42—H42D	113.0
O10—C55—C54	115.8 (2)	H42A—C42—H42B	106.4
O10—C55—C56	124.4 (2)	H42C—C42—H42D	110.4
C54—C55—C56	119.8 (2)	C42—C43A—H43A	111.8
C55—C56—C57	121.4 (2)	C42—C43A—H43B	111.8
C52—C57—C56	118.5 (2)	C44A—C43A—H43A	111.8
C52—C57—C58	118.9 (2)	C44A—C43A—H43B	111.8
C56—C57—C58	122.6 (2)	H43A—C43A—H43B	109.5
C49—C58—C57	121.2 (2)	C42—C43B—H43C	108.8
C26—O4—H4O	109.5	C42—C43B—H43D	108.8
C25—O5—H5O	109.5	C44B—C43B—H43C	108.8
C54—O9—H9O	109.5	C44B—C43B—H43D	108.8
C55—O10—H10O	109.5	H43C—C43B—H43D	107.7
C2—C1—H1	120.2	C43A—C44A—H44A	109.5
C6—C1—H1	119.2	C43A—C44A—H44B	109.5
C1—C2—H2	118.7	C43A—C44A—H44C	109.5
C3—C2—H2	120.7	H44A—C44A—H44B	109.5
C2—C3—H3	122.2	H44A—C44A—H44C	109.5
C4—C3—H3	118.0	H44B—C44A—H44C	109.5
C3—C4—H4	120.2	C43B—C44B—H44D	109.5
C5—C4—H4	119.8	C43B—C44B—H44E	109.5
C4—C5—H5	119.9	C43B—C44B—H44F	109.5
C6—C5—H5	118.8	H44D—C44B—H44E	109.5
N1—C7—H7A	108.8	H44D—C44B—H44F	109.5
N1—C7—H7B	108.4	H44E—C44B—H44F	109.5
C6—C7—H7A	108.3	N2—C45—H45A	108.6
C6—C7—H7B	107.6	N2—C45—H45B	108.5
H7A—C7—H7B	107.3	C46—C45—H45A	108.2
N1—C8—H8A	107.7	C46—C45—H45B	107.6
N1—C8—H8B	108.1	H45A—C45—H45B	107.0
C9—C8—H8A	108.9	C45—C46—H46A	109.3
C9—C8—H8B	109.1	C45—C46—H46B	109.9
H8A—C8—H8B	106.8	C47—C46—H46A	109.2
C8—C9—H9A	110.5	C47—C46—H46B	110.1
C8—C9—H9B	110.5	H46A—C46—H46B	107.5
C10—C9—H9A	109.4	C46—C47—H47A	108.8
C10—C9—H9B	110.4	C46—C47—H47B	109.8
H9A—C9—H9B	107.7	C48—C47—H47A	107.2
C9—C10—H10A	109.2	C48—C47—H47B	107.8
C9—C10—H10B	109.6	H47A—C47—H47B	106.2
C11—C10—H10A	106.5	C47—C48—H48A	111.5
C11—C10—H10B	107.0	C47—C48—H48B	109.3
H10A—C10—H10B	107.7	C47—C48—H48C	108.9
C10—C11—H11A	109.8	H48A—C48—H48B	109.5
C10—C11—H11B	109.5	H48A—C48—H48C	109.7
C10—C11—H11C	109.5	H48B—C48—H48C	107.8
H11A—C11—H11B	108.8	C49—C50—H50	120.1
H11A—C11—H11C	109.7	C51—C50—H50	119.7
H11B—C11—H11C	109.4	C50—C51—H51	118.8
N1—C12—H12A	108.1	C52—C51—H51	119.5
N1—C12—H12B	108.4	C52—C53—H53	119.3
C13—C12—H12A	107.5	C54—C53—H53	119.3
C13—C12—H12B	107.6	C55—C56—H56	118.7
H12A—C12—H12B	106.8	C57—C56—H56	119.8
C12—C13—H13A	109.8	C49—C58—H58	119.6
C12—C13—H13B	109.9	C57—C58—H58	119.2
			
O6—S1—C49—C50	−90.7 (3)	C22—C23—C28—C27	178.9 (3)
O6—S1—C49—C58	89.0 (3)	C22—C23—C28—C29	−0.6 (4)
O7—S1—C49—C50	148.2 (2)	C24—C23—C28—C27	−0.3 (4)
O7—S1—C49—C58	−32.1 (3)	C24—C23—C28—C29	−179.9 (3)
O8—S1—C49—C50	28.3 (3)	C28—C23—C24—C25	0.4 (4)
O8—S1—C49—C58	−152.0 (2)	C23—C24—C25—O5	178.4 (3)
O1—S2—C20—C21	−87.9 (3)	C23—C24—C25—C26	−0.3 (4)
O1—S2—C20—C29	89.1 (3)	O5—C25—C26—O4	0.1 (3)
O2—S2—C20—C21	33.1 (3)	O5—C25—C26—C27	−178.7 (2)
O2—S2—C20—C29	−149.9 (2)	C24—C25—C26—O4	178.9 (3)
O3—S2—C20—C21	151.8 (2)	C24—C25—C26—C27	0.1 (3)
O3—S2—C20—C29	−31.3 (3)	O4—C26—C27—C28	−178.7 (2)
C7—N1—C8—C9	66.4 (4)	C25—C26—C27—C28	−0.0 (4)
C8—N1—C7—C6	51.8 (4)	C26—C27—C28—C23	0.1 (3)
C7—N1—C12—C13	−64.1 (3)	C26—C27—C28—C29	179.7 (3)
C12—N1—C7—C6	−65.0 (4)	C23—C28—C29—C20	0.2 (4)
C7—N1—C16—C17	−179.5 (3)	C27—C28—C29—C20	−179.3 (3)
C16—N1—C7—C6	173.4 (3)	C31—C30—C35—C34	−2.0 (7)
C8—N1—C12—C13	175.2 (3)	C31—C30—C35—C36	−178.9 (4)
C12—N1—C8—C9	−172.7 (3)	C35—C30—C31—C32	0.8 (8)
C8—N1—C16—C17	−58.1 (4)	C30—C31—C32—C33	0.1 (7)
C16—N1—C8—C9	−51.8 (4)	C31—C32—C33—C34	0.3 (7)
C12—N1—C16—C17	58.8 (4)	C32—C33—C34—C35	−1.6 (8)
C16—N1—C12—C13	54.2 (4)	C33—C34—C35—C30	2.4 (7)
C36—N2—C37—C38	67.2 (4)	C33—C34—C35—C36	179.2 (4)
C37—N2—C36—C35	52.3 (4)	C30—C35—C36—N2	−104.5 (4)
C36—N2—C41—C42	−61.7 (4)	C34—C35—C36—N2	78.8 (5)
C41—N2—C36—C35	−64.9 (4)	N2—C37—C38—C39	179.8 (3)
C36—N2—C45—C46	−177.6 (3)	C37—C38—C39—C40	69.5 (6)
C45—N2—C36—C35	174.1 (3)	N2—C41—C42—C43A	149.6 (5)
C37—N2—C41—C42	177.4 (3)	N2—C41—C42—C43B	176.0 (5)
C41—N2—C37—C38	−171.5 (3)	C41—C42—C43A—C44A	70.8 (9)
C37—N2—C45—C46	−56.1 (4)	C41—C42—C43B—C44B	176.3 (10)
C45—N2—C37—C38	−51.3 (4)	N2—C45—C46—C47	177.5 (3)
C41—N2—C45—C46	60.7 (4)	C45—C46—C47—C48	−174.4 (4)
C45—N2—C41—C42	56.5 (4)	S1—C49—C50—C51	−177.7 (2)
C2—C1—C6—C5	−1.6 (6)	S1—C49—C58—C57	179.8 (2)
C2—C1—C6—C7	−178.0 (4)	C50—C49—C58—C57	−0.5 (5)
C6—C1—C2—C3	−0.0 (7)	C58—C49—C50—C51	2.7 (5)
C1—C2—C3—C4	1.2 (8)	C49—C50—C51—C52	−1.7 (5)
C2—C3—C4—C5	−0.7 (8)	C50—C51—C52—C53	178.3 (3)
C3—C4—C5—C6	−0.9 (7)	C50—C51—C52—C57	−1.4 (5)
C4—C5—C6—C1	2.1 (6)	C51—C52—C53—C54	178.1 (3)
C4—C5—C6—C7	178.4 (4)	C51—C52—C57—C56	−176.6 (3)
C1—C6—C7—N1	−101.7 (4)	C51—C52—C57—C58	3.4 (4)
C5—C6—C7—N1	82.0 (4)	C53—C52—C57—C56	3.8 (4)
N1—C8—C9—C10	178.8 (3)	C53—C52—C57—C58	−176.2 (3)
C8—C9—C10—C11	67.1 (7)	C57—C52—C53—C54	−2.3 (5)
N1—C12—C13—C14	175.9 (3)	C52—C53—C54—O9	179.8 (3)
C12—C13—C14—C15	171.8 (4)	C52—C53—C54—C55	−1.6 (5)
N1—C16—C17—C18	173.7 (3)	O9—C54—C55—O10	1.9 (4)
C16—C17—C18—C19	−175.0 (4)	O9—C54—C55—C56	−177.3 (3)
S2—C20—C21—C22	176.1 (2)	C53—C54—C55—O10	−176.9 (3)
S2—C20—C29—C28	−176.5 (2)	C53—C54—C55—C56	3.9 (5)
C21—C20—C29—C28	0.5 (5)	O10—C55—C56—C57	178.5 (3)
C29—C20—C21—C22	−0.8 (5)	C54—C55—C56—C57	−2.4 (5)
C20—C21—C22—C23	0.4 (5)	C55—C56—C57—C52	−1.5 (4)
C21—C22—C23—C24	179.5 (3)	C55—C56—C57—C58	178.5 (3)
C21—C22—C23—C28	0.3 (5)	C52—C57—C58—C49	−2.6 (4)
C22—C23—C24—C25	−178.8 (3)	C56—C57—C58—C49	177.5 (3)

Symmetry codes: (i) −*x*+1, −*y*+2, −*z*+1; (ii) *x*−1, *y*+1, *z*; (iii) −*x*+2, −*y*+1, −*z*; (iv) *x*+1, *y*−1, *z*; (v) −*x*+1, −*y*+1, −*z*+1; (vi) *x*+1, *y*, *z*; (vii) −*x*, −*y*+2, −*z*+1; (viii) −*x*+2, −*y*+1, −*z*+1; (ix) −*x*+1, −*y*+1, −*z*; (x) −*x*+1, −*y*+2, −*z*; (xi) *x*, *y*, *z*−1.

### Hydrogen-bond geometry (Å, °)

**Table:**

*D*—H···*A*	*D*—H	H···*A*	*D*···*A*	*D*—H···*A*
O4—H4O···O3^i^	0.82	1.85	2.670 (3)	174
O5—H5O···O8^ii^	0.82	1.83	2.625 (3)	163
O9—H9O···O2	0.82	1.85	2.650 (3)	165
O10—H10O···O6^iii^	0.82	1.93	2.705 (3)	158

Symmetry codes: (i) −*x*+1, −*y*+2, −*z*+1; (ii) *x*−1, *y*+1, *z*; (iii) −*x*+2, −*y*+1, −*z*.
